# A Connecting Link between Hyaluronan Synthase 3-Mediated Hyaluronan Production and Epidermal Function

**DOI:** 10.3390/ijms23052424

**Published:** 2022-02-22

**Authors:** Yukiko Ota, Hiroyuki Yoshida, Yoko Endo, Tetsuya Sayo, Yoshito Takahashi

**Affiliations:** Biological Science Research, Kao Corporation, 5-3-28 Kotobuki-cho, Odawara 250-0002, Japan; oota.yukiko@kao.com (Y.O.); endou.youko1@kao.com (Y.E.); sayou.tetsuya@kao.com (T.S.); takahashi.yoshito@kao.com (Y.T.)

**Keywords:** hyaluronan, hyaluronan synthase 3, epidermis, proliferation, differentiation

## Abstract

Hyaluronan (HA), an essential component of the extracellular matrix of the skin, is synthesized by HA synthases (HAS1-3). To date, epidermal HA has been considered a major player in regulating cell proliferation and differentiation. However, a previous study reported that depletion of epidermal HA by *Streptomyces* hyaluronidase (St-HAase) has no influence on epidermal structure and function. In the present study, to further explore roles of epidermal HA, we examined effects of siRNA-mediated knockdown of *HAS3*, as well as conventional HA-depletion methods using St-HAase and 4-methylumbelliferone (4MU), on epidermal turnover and architecture in reconstructed skin or epidermal equivalents. Consistent with previous findings, HA depletion by St-HAase did not have a substantial influence on the epidermal architecture and turnover in skin equivalents. 4MU treatment resulted in reduced keratinocyte proliferation and epidermal thinning but did not seem to substantially decrease the abundance of extracellular HA. In contrast, siRNA-mediated knockdown of *HAS3* in epidermal equivalents resulted in a significant reduction in epidermal HA content and thickness, accompanied by decreased keratinocyte proliferation and differentiation. These results suggest that HAS3-mediated HA production, rather than extracellularly deposited HA, may play a role in keratinocyte proliferation and differentiation, at least in the developing epidermis in reconstructed epidermal equivalents.

## 1. Introduction

Hyaluronan (HA) is a linear and extra-large glycosaminoglycan that is composed of disaccharide repeats of glucuronic acid and *N*-acetylglucosamine (GlcNAc), and is abundant in the extracellular matrix of connective tissues. HA is widely distributed throughout the body, and more than 50% of total body HA content is present in the skin, contributing to skin hydration and elasticity owing to its extraordinary water-holding capacity [1,2]. In the epidermis, HA is primarily localized in the extracellular space from the basal to the granular layer [3], and enables cells to facilitate the exchange of waste and nutrients [2]. HA is synthesized in the plasma membrane by three different hyaluronan synthases (HAS1-3), which utilize cytosolic UDP-GlcNAc and UDP-glucuronic acid as substrates [4,5,6,7,8]. Expression patterns of *HAS1-3* mRNAs depend on cell type, and we and other research groups have previously reported that *HAS3* plays a crucial role in HA production in normal human epidermal keratinocytes [9,10,11,12,13].

Epidermal function and structure depend on a tightly regulated balance between keratinocyte proliferation and differentiation [14], and multiple lines of evidence have suggested that HA plays an important role in regulating epidermal integrity by controlling cell proliferation and differentiation. For example, enhanced epidermal HA stimulated by keratinocyte growth factor, epidermal growth factor, or retinoic acid is correlated with increased cell proliferation [15,16,17,18]. Conversely, the reduction in epidermal HA by the HA synthesis inhibitor 4-methylumbelliferone (4MU) in organotypic cultures of rat keratinocytes or by a deficiency in the cell surface HA receptor CD44 in mice led to decreased keratinocyte proliferation and differentiation, respectively [19,20]. Furthermore, we recently reported that enhanced epidermal HA production mediated by 1-ethyl-β-*N*-acetylglucosaminide, a new inducer of epidermal HA production [21,22], resulted in epidermal thickening by accelerating keratinocyte proliferation and differentiation in reconstructed human skin equivalents [23]. Therefore, epidermal HA is generally considered to boost epidermal proliferation and differentiation. However, contradictory findings have also been reported on the relationship between epidermal HA and keratinocyte behaviors; the depletion of epidermal HA by *Streptomyces* hyaluronidase (St-HAase), which specifically degrades extracellular HA, had no effect on proliferation and differentiation processes in reconstructed human epidermal equivalents [24]. Therefore, the biological functions of epidermal HA are not fully understood, and new approaches are required to further elucidate the functions of epidermal HA.

In the present study, in addition to using conventional HA-depletion strategies such as St-HAase and 4MU, we performed *HAS3* silencing with siRNAs to deplete endogenous epidermal HA production in reconstructed human epidermal equivalents. We found that St-HAase-mediated HA depletion did not alter *HAS3* mRNA expression, epidermal structure, or keratinocyte proliferation or differentiation. However, the reduction in epidermal HA caused by decreased HAS3-mediated HA production led to epidermal thinning by decreasing keratinocyte proliferation and differentiation. Our data in the present study provides evidence that epidermal HA production mediated by HAS3 may play a role in epidermal organization, by regulating keratinocyte proliferation and differentiation.

## 2. Results

### 2.1. Streptomyces Hyaluronidase Does Not Change Epidermal Thickness, Keratinocyte Proliferation and Differentiation, or HAS3 mRNA Expression in Reconstructed Human Skin Equivalents

**Figure 1:**
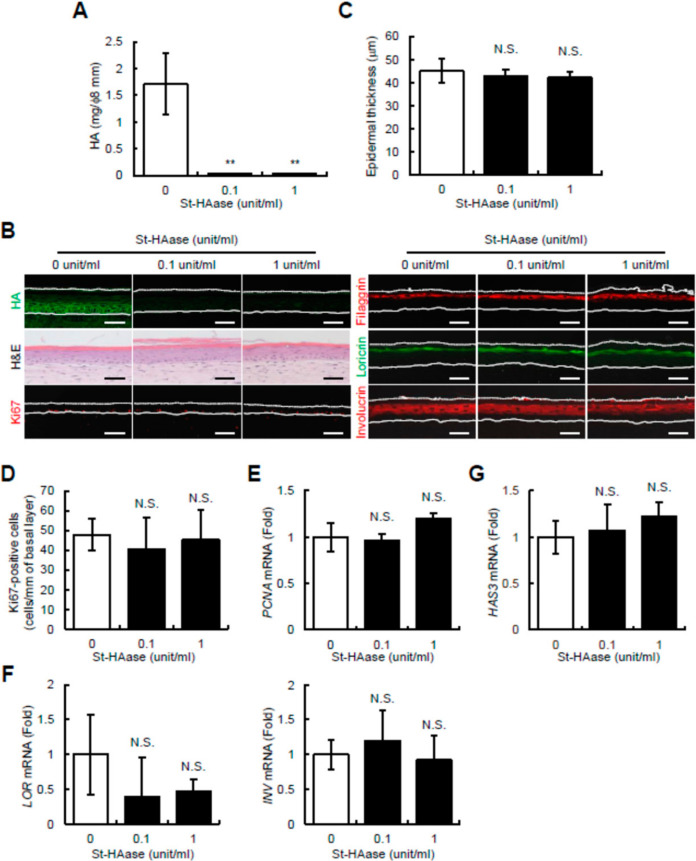
The effects of *Streptomyces* hyaluronidase (St-HAase) on epidermal HA, structure, and turnover in reconstructed human skin equivalents. (**A**) Amount of HA in St-HAase-treated skin equivalents. The skin equivalents were treated with St-HAase (0, 0.1, or 1 unit/mL) for 9 days. The epidermal layer was separated, and HA was quantified by ELISA. Values are expressed as the mean ± SD (*n* = 3) and shown as mg/φ8 mm. The Dunnett’s test was used for statistical analysis. ** *p* < 0.01. (**B**) Representative images of HA, H&E, Ki67, filaggrin, loricrin, and involucrin staining. The skin equivalents cultured with St-HAase (0, 0.1, or 1 unit/mL) for 5 days (for Ki67 staining) or 9 days (for HA, H&E, filaggrin, loricrin, or involucrin staining) were collected. The sections were stained for HA (*green*) or morphology (H&E) and immunostained for Ki67 (red), filaggrin (red), loricrin (green), or involucrin (red). Dotted lines represent the skin’s surface and the solid lines indicate the border between the epidermis and dermis. Scale bars = 50 μm. (**C**) Epidermal thickness of St-HAase-treated skin equivalents. Skin equivalents were collected 9 days after St-HAase treatment (0, 0.1, or 1 unit/mL), and epidermal thickness was assessed. Values are expressed as the mean ± SD (*n* = 3) and shown as μm. The Dunnett’s test was used for statistical analysis. N.S., not significant. (**D**) The Ki67-positive cells in St-HAase-treated skin equivalents. The skin equivalents were treated with St-HAase (0, 0.1, or 1 unit/mL) for 5 days, and the number of Ki67-positive cells in the epidermis was counted. Values are expressed as the mean ± SD (*n* = 3) and shown as cells/mm of the basal layer. The Dunnett’s test was used for statistical analysis. N.S., not significant. (**E**,**F**) *PCNA* (**E**), *LOR*, and *INV* (**F**) mRNA expression in St-HAase-treated skin equivalents. The expression levels of *PCNA* mRNA 5 days (**E**) and *LOR* and *INV* mRNAs 9 days (**F**) after treatment of skin equivalents with St-HAase (0, 0.1, or 1 unit/mL) were evaluated by quantitative real-time PCR. Values are expressed as the mean ± SD (*n* = 3) and shown as x-fold increases in mRNA expression relative to control samples. The Dunnett’s test was used for statistical analysis. N.S., not significant. (**G**) Expression level of *HAS3* mRNA in St-HAase-treated skin equivalents. The expression level of *HAS3* mRNA in skin equivalents treated with St-HAase (0, 0.1, or 1 unit/mL) for 9 days was measured by quantitative real-time PCR. The Dunnett’s test was used for statistical analysis. N.S., not significant.

Hyaluronidase from *Streptomyces* (St-HAase) is widely used to selectively digest HA and to remove it from tissues. Although previous studies have indicated a strong interaction between epidermal HA and keratinocyte proliferation or differentiation [15,16,17,18,19,20,23], St-HAase treatment was reported to have no effect on keratinocyte proliferation and differentiation in reconstructed human epidermal equivalents [24]. In the present study, to examine the discrepancy among previous studies, we investigated the effect of St-HAase on epidermal structure and turnover, that is, keratinocyte proliferation and differentiation processes, in reconstructed human skin equivalents with a fibroblast-populated collagen matrix. During epidermal reconstruction from day 0 (lifted to the air–liquid interface) until day 9 (fully differentiated and stratified), skin equivalents were treated in the absence or presence of St-HAase (0.1, or 1 unit/mL). As shown in Figure 1A, when the epidermal HA amount was determined after the detachment of the epidermis from the dermis, the amount of epidermal HA (mg per 8 mm diameter epidermal pieces) drastically decreased in St-HAase-treated tissues compared to the non-treated control tissues. The histological localization of HA, via HA staining using HA binding protein (HABP), showed that extracellular HA staining in the basal to granular layers was depleted by the St-HAase treatment (Figure 1B), suggesting that almost all epidermal HA was efficiently digested by the St-HAase treatment. Under these conditions, hematoxylin and eosin (H&E) staining showed that the epidermal thickness of St-HAase-treated skin equivalents was unchanged compared to that of non-treated control samples (Figure 1B). Quantitative analysis of the epidermal thickness confirmed these histological observations by showing that the epidermal thickness of St-HAase-treated skin equivalents was not significantly changed compared to that of the non-treated control culture (Figure 1C). We then examined the effect of St-HAase on epidermal turnover in skin equivalents. As shown in Figure 1B, immunohistochemical staining of the proliferation marker Ki67 demonstrated that Ki67-positive cells were equally observed in the basal layer of the control and in St-HAase-treated skin equivalents. By counting the number of Ki67-positive cells, we confirmed that there was no significant difference in the number of Ki67-positive cells between the control and St-HAase-treated skin equivalents (Figure 1D). We further revealed that the mRNA expression of another proliferation marker, *PCNA*, was at the same level between the control and St-HAase-treated skin samples (Figure 1E). We then performed immunostaining of differentiation markers such as filaggrin, loricrin, and involucrin, and no difference in staining intensity was observed between the control and St-HAase-treated skin equivalents (Figure 1B). We also obtained data that there was no significant difference in mRNA expression of *LOR* (*loricrin*) and *INV* (*involucrin*), with or without St-HAase treatment (Figure 1F). These results were in agreement with previous findings [24]; that is, St-HAase treatment almost completely removed HA from the epidermis, but HA depletion is unlikely to have a substantial influence on epidermal architecture and turnover. Furthermore, we also assessed the mRNA expression of *HAS3*, which plays a dominant role in epidermal HA production among *HAS1-3* in skin equivalents [23], and observed no difference in *HAS3* mRNA expression in the control and St-HAase-treated skin equivalents (Figure 1G). *HAS1* and *HAS2* mRNA expression levels were not significantly altered by St-HAase treatment (data not shown). These results suggest that St-HAase treatment directly digested pre-existing and newly secreted HA in the tissues but did not change *HAS3* mRNA expression, that is, the ability of keratinocytes to produce epidermal HA.

### 2.2. Presence of 4-Methylumbelliferone Decreases Epidermal Thickness and Keratinocyte Proliferation in Reconstructed Human Skin Equivalents

**Figure 2:**
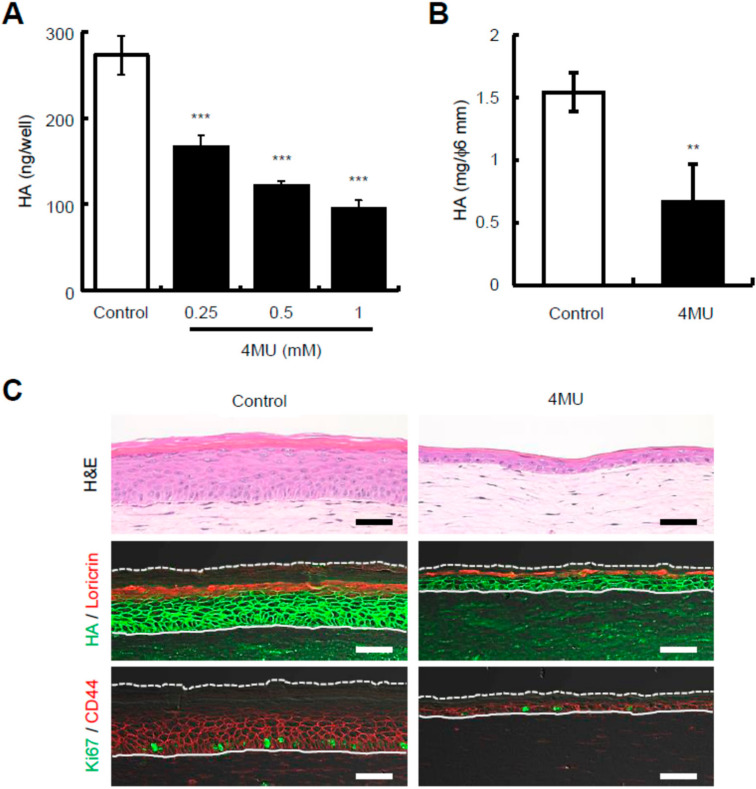
The effect of 4-methylumbelliferone (4MU) on HA production in cultured normal human skin keratinocytes, and on epidermal HA, structure, and turnover in reconstructed human skin equivalents. (**A**) HA production in 4MU-treated keratinocytes. Cultured keratinocytes were treated with 4MU (0, 0.25, 0.5, or 1 mM) for 2 days. HA concentrations in the culture media were quantified by ELISA. Values are expressed as means ± SD (*n* = 3) and are shown as ng/well. The Dunnett’s test was used for statistical analysis. *** *p* < 0.001. (**B**) HA amounts in 4MU-treated skin equivalents. Skin equivalents were treated with 4MU (0 or 0.5 mM) for 9 days. The epidermal layer was detached, and HA was determined by ELISA. Values are expressed as the mean ± SD (*n* = 3) and shown as mg/φ6 mm. The Student’s *t*-test was used for statistical analysis. ** *p* < 0.01. (**C**) Representative images of H&E, HA, loricrin, Ki67, and CD44 staining. The skin equivalents cultured with 4MU (0 or 0.5 mM) for 9 days were collected. The sections were stained for morphology (H&E) or HA (*green*) and immunostained for loricrin (red), Ki67 (green), or CD44 (red). Dotted lines represent the skin surface and the solid lines indicate the border between the epidermis and dermis. Scale bars = 50 μm.

To confirm the observations obtained using St-HAase, 4-methylumbelliferone (4MU), a classic HA synthesis inhibitor, was used to manipulate epidermal HA in reconstructed skin equivalents. Previously, 4MU was utilized to remove HA in reconstructed epidermal equivalents, but it was less efficient than St-HAase [24]. Therefore, we first checked the effect of 4MU on HA production in the monolayer culture of normal human epidermal keratinocytes, and confirmed that HA content in the culture media was dose-dependently decreased by 4MU treatment (0, 0.25, 0.5, or 1 mM) (Figure 2A). We then treated reconstructed human skin equivalents with 0.5 mM 4MU, and showed that a 9-day treatment with 4MU partially, but significantly, decreased epidermal HA amounts (mg per 6 mm diameter epidermal pieces) in reconstructed skin equivalents (Figure 2B). Under these conditions, H&E staining demonstrated that the epidermal thickness of 4MU-treated skin equivalents decreased drastically (Figure 2C). Immunohistochemical studies revealed that the distribution pattern of loricrin appeared to be unchanged (Figure 2C), but Ki67-positive cells were markedly decreased in 4MU-treated skin equivalents compared to the control culture (Figure 2C). On the other hand, it should be noted that the staining intensity of epidermal HA in 4MU-treated skin equivalents seemed to only be slightly decreased compared to that in control skin equivalents (Figure 2C), suggesting that HA depletion by 4MU (Figure 2B) may be more strongly attributed to epidermal thinning rather than the inhibition of HA synthesis. We also found that the overlapping expression pattern of the cell surface HA receptor CD44, which is known to contribute to the regulation of keratinocyte proliferation and differentiation [25], with HA appeared to remain unchanged by 4MU (Figure 2C). Therefore, we could not exclude the possibility that 4MU may affect epidermal structure and proliferation independently of the HA or HA-CD44 axis. Thus, it is uncertain whether epidermal HA is associated with epidermal structure and keratinocyte physiology after 4MU treatment.

### 2.3. Reduction of HAS3-Mediated HA Production Decreases Epidermal Thickness and Keratinocyte Proliferation and Differentiation in Reconstructed Human Epidermal Equivalents

**Figure 3:**
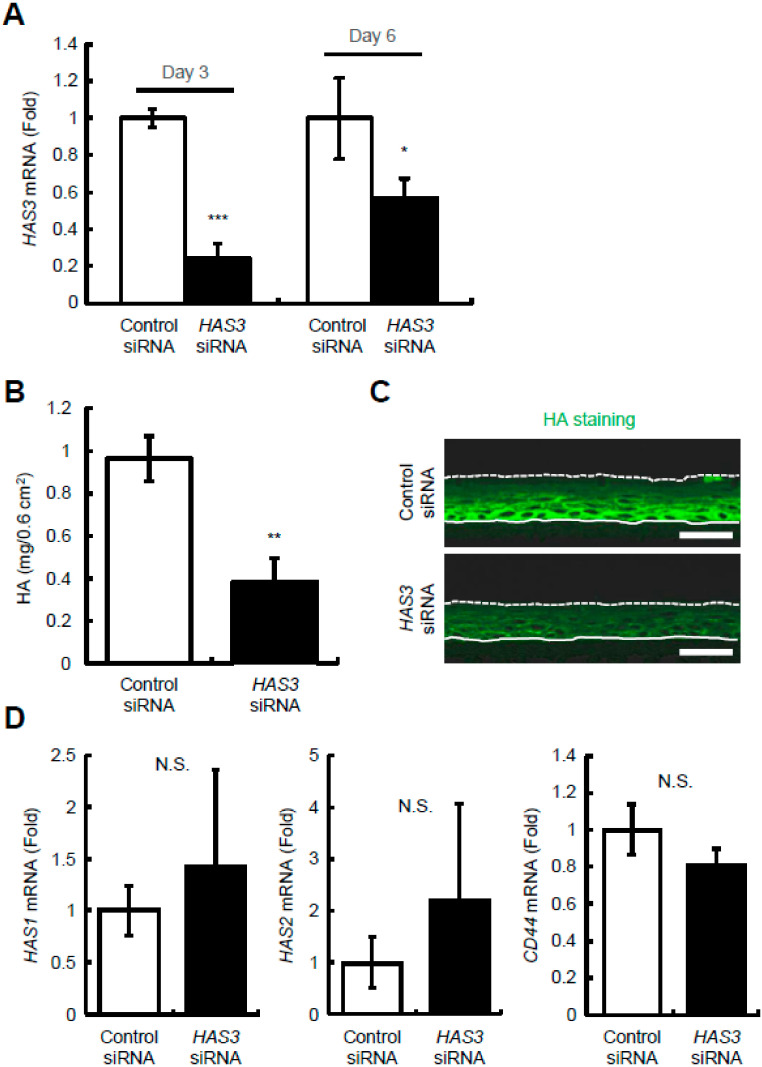
The effect of *HAS3* knockdown on epidermal HA, and *HAS1-3* and *CD44* mRNA expression in reconstructed human epidermal equivalents. (**A**) Knockdown efficiency for *HAS3*. The expression levels of *HAS3* mRNA 3 and 6 days after treatment of keratinocytes with siRNA (s6460) were evaluated by quantitative real-time PCR. As for controls, keratinocytes were transfected with control non-silencing siRNA (control siRNA). Values are expressed as means ± SD (*n* = 3) and shown as x-fold increases in mRNA expression relative to control samples. The Student’s *t*-test was used for statistical analysis. * *p* < 0.05; *** *p* < 0.001. Representative data for two siRNAs are shown. (**B**,**C**) HA amounts (**B**) and distribution (**C**) in *HAS3*-knocked down epidermal equivalents. Epidermal equivalents were collected 6 days after treatment of keratinocytes with siRNA, and HA was quantified by ELISA (**B**). Values are expressed as the mean ± SD (*n* = 3) and shown as mg/0.6 cm^2^. The Student’s *t*-test was used for statistical analysis. ** *p* < 0.01. The sections of samples collected after 6 days were stained for HA (green) (**C**). (**D**) Knockdown specificity for *HAS3*. The expression levels of *HAS1-2* and *CD44* mRNA 3 days after treatment of keratinocytes with *HAS3* siRNA (s6460) were evaluated by quantitative real-time PCR. As for controls, keratinocytes were transfected with control non-silencing siRNA (control siRNA). Values are expressed as the mean ± SD (*n* = 3) and shown as x-fold increases in mRNA expression relative to control samples. The Student’s *t*-test was used for statistical analysis. N.S., not significant.

To further explore the role of HA in the epidermis, we performed siRNA-mediated gene silencing of *HAS3* in reconstructed human epidermal equivalents. We first assessed the relative amount of *HAS* mRNA transcripts in epidermal equivalents using quantitative real-time PCR, and found that *HAS3* was the prominent isoform, accounting for approximately 98% of *HAS* transcripts (data not shown). We then prepared reconstructed epidermal equivalents with human keratinocytes treated with *HAS3* siRNAs and showed that the expression of *HAS3* mRNA was knocked down to approximately 25% (Day 3) and 60% (Day 6) (Figure 3A), and HA amounts were significantly decreased to approximately 41% (Day 6) after siRNA transfection compared to control levels (Figure 3B). As expected, HA staining showed that HA signals between cells were much weaker in *HAS3* knocked-down epidermal equivalents than in the control culture (Figure 3C). Notably, there was no significant difference in the expression of *HAS1*, *HAS2*, and *CD44* mRNAs between the control and *HAS3* knocked-down epidermal equivalents (Figure 3D), suggesting that effective, long-term, and specific knockdown of *HAS3* mRNA expression was achieved.

**Figure 4:**
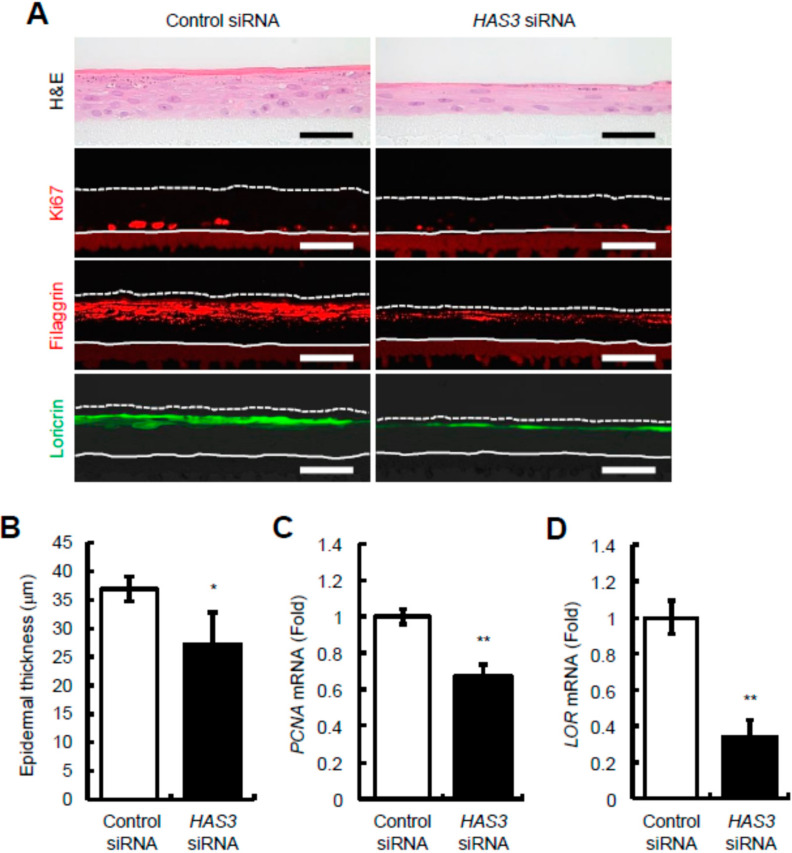
The effect of *HAS3* knockdown on epidermal structure and turnover in reconstructed human epidermal equivalents. (**A**) Representative images of H&E, Ki67, filaggrin, and loricrin staining. Epidermal equivalents were collected 6 days after treatment of keratinocytes with *HAS3* siRNA (s6460). The sections were stained for morphology (H&E) and immunostained for Ki67 (red), filaggrin (red), or loricrin (green). As for controls, keratinocytes were transfected with control non-silencing siRNA (control siRNA). Dotted lines represent the skin’s surface and the solid lines indicate the border between the epidermis and dermis. Scale bars = 50 μm. (**B**) Epidermal thickness of *HAS3* knocked-down epidermal equivalents. Epidermal equivalents were collected 6 days after treatment of keratinocytes with *HAS3* siRNA (s6460), and epidermal thickness was assessed. Values are expressed as means ± SD (*n* = 3) and shown as μm. The Student’s *t*-test was used for statistical analysis. * *p* < 0.05. (**C**,**D**) *PCNA* (**C**) and *LOR* (**D**) mRNA expression in *HAS3* knocked-down epidermal equivalents. The expression levels of *PCNA* mRNA 3 days (**C**) and *LOR* mRNA 6 days (**D**) after treatment of keratinocytes with *HAS3* siRNA (s6460) were evaluated by quantitative real-time PCR. As for controls, keratinocytes were transfected with control non-silencing siRNA (control siRNA). Values are expressed as the mean ± SD (*n* = 3) and shown as x-fold increases in mRNA expression relative to control samples. The Student’s *t*-test was used for statistical analysis. ** *p* < 0.01.

To investigate the influence of *HAS3* knockdown-mediated HA depletion on epidermal structure and keratinocyte behavior, we conducted H&E staining and showed that the epidermal thickness of *HAS3* knocked-down epidermal equivalents decreased compared to control samples (Figure 4A). These histological observations were confirmed by quantitative analysis of epidermal thickness, which showed that the epidermal thickness of *HAS3* knocked-down epidermal equivalents was significantly decreased compared with that of the control culture (Figure 4B). Immunohistochemical staining demonstrated that Ki67-positive cells were sparsely distributed in the basal layer of *HAS3* knocked-down epidermal equivalents compared to control epidermal equivalents (Figure 4A), and that signals of filaggrin and loricrin in the granular layer decreased in *HAS3* knocked-down epidermal equivalents compared with control samples (Figure 4A). We also showed that mRNA expression of *PCNA* (Figure 4C) and *LOR* (Figure 4D) significantly decreased in *HAS3* knocked-down epidermal equivalents compared to control equivalents. These results strongly indicated that the reduction in HAS3-mediated HA production decreased keratinocyte proliferation and differentiation, thereby inducing epidermal thinning. Thus, these findings suggest that HAS3-mediated HA production may play a role in epidermal organization, at least in the developing epidermis, by regulating fundamental cellular processes including cell proliferation and differentiation.

## 3. Discussion

In the present study, we investigated the role of HA in regulating epidermal function and structure using three different experimental methods: HA digestion by St-HAase, inhibition of HA synthesis by 4MU, and HA depletion by siRNA-mediated HAS3 knockdown. Among them, 4MU treatment resulted in the most prominent epidermal thinning accompanied by reduced keratinocyte proliferation, which is generally consistent with previous findings that show that 4MU decreased keratinocyte proliferation accompanied by epidermal thinning in organotypic cultures of rat keratinocytes or reconstructed human epidermal equivalents [19,24]. 4MU is conventionally used to inhibit HA synthesis by decreasing the intracellular pool of UDP-glucuronic acid, a substrate for HA production by HAS enzymes [26,27,28], and we confirmed that 4MU decreases HA production in cultured human skin keratinocytes, as well as HA amounts (per epidermal area) in reconstructed human skin equivalents. However, since HA staining showed that 4MU did not substantially decrease the abundance of extracellular HA in the epidermis of skin equivalents, the relationship between epidermal HA and epidermal thinning and decreased keratinocyte proliferation remained inconclusive. Moreover, the diverse pharmacological activities of 4MU have been reported, in which 4MU decreases the proliferation rate of smooth muscle cells [29] as well as many cancer cells [30,31,32,33,34], and induces apoptosis [35], all of which are not systematically linked to HA. Therefore, although further studies are needed, it seems plausible to speculate that 4MU-mediated epidermal thinning and decreased proliferation are also dependent on mechanisms other than epidermal HA. In the present study, we used H&E images and imaging software to measure the epidermal thickness of 4MU-treated skin equivalents. However, optical coherence tomography, a real-time and non-invasive imaging technique, can also be utilized to monitor the thickness change of the epidermis more easily.

One of the interesting findings of this study was that differences between the effects produced by St-HAase and siRNA-mediated HAS3 knockdown were observed; St-HAase-mediated HA depletion did not apparently change epidermal turnover and structure, whereas HAS3 siRNA-mediated HA reduction resulted in epidermal thinning by decreasing keratinocyte proliferation and differentiation. Through quantitative and histological analyses, we demonstrated that the two different HA-depleting strategies commonly decreased extracellular HA abundance in the epidermis, and thus, extracellular HA levels were unlikely to be related to epidermal morphology and cell proliferation and differentiation. In contrast, St-HAase treatment directly digested tissue HA without affecting *HAS3* mRNA expression, whereas siRNA-mediated *HAS3* silencing decreased HA levels by suppressing HA production mediated by HAS3. Therefore, it is tempting to speculate that HAS3-mediated HA production, rather than the extracellular level of epidermal HA, may be linked to epidermal organization and function, including keratinocyte proliferation and differentiation, at least in the developing epidermis in reconstructed epidermal equivalents. This notion is supported by our previous findings that 1-ethyl-β-*N*-acetylglucosaminide, an inducer of HAS3-mediated HA production [21], caused epidermal thickening by accelerating keratinocyte proliferation and differentiation [22,23]. However, since small HA oligosaccharides, which are known to induce keratinocyte proliferation and differentiation [36,37], could not be measured with the HA ELISA assay, we cannot exclude the possibility that changes in HA size by St-HAase treatment may influence epidermal organization and architecture. However, it is still uncertain how HAS3-mediated HA production is involved in keratinocyte behavior. Previous studies have reported that HA metabolism regulates the cytosolic concentration of UDP-GlcNAc, a substrate not only for HA, but also for *o*-linked GlcNAc modification and other glycosaminoglycans, including keratan sulfate, heparan sulfate, and dermatan/chondroitin [38,39]. Of note, protein modification by *o*-linked GlcNAc, versican, a large chondroitin sulfate proteoglycan, glypican-1 and syndecan-1, and heparan sulfate cell membrane proteoglycans, all of which are expressed in the epidermis [40], are known to be involved in cell proliferation and/or the differentiation of various types of cells [41,42,43,44,45,46,47]. Therefore, one can speculate that HAS3-mediated HA production may regulate keratinocyte proliferation and differentiation by affecting intracellular UDP-GlcNAc levels. Another possibility is that newly produced HA from HAS3 might immediately interact with cell surface HA receptors, including CD44, and regulate keratinocyte behavior, which should be addressed in future studies.

A potential limitation of the current study is that we obtained data only from in vitro reconstructed human skin or epidermal equivalent studies. Previously, knockout mice lacking functional HAS1, HAS3, or both were reported to show unaltered keratinocyte proliferation even during wound healing conditions [48,49,50]. In addition, HA fragments have been reported to activate keratinocytes to produce β-defensin 2, an antimicrobial peptide, via toll-like receptor (TLR) 2 and TLR4 [51], which might in turn influence the skin microbiome necessary for cutaneous homeostasis. Therefore, further detailed studies are needed to elucidate the in vivo relevance of HAS3-mediated epidermal HA production on epidermal function. Additionally, the relative contributions of HAS1, HAS2, and HAS3 in epidermal HA production remain controversial. Specifically, HAS1 was reported to be the dominant isoenzyme in human keratinocyte monolayers and reconstructed human epidermal equivalents [13], whereas HAS2 was significantly expressed in biopsies of human skin [13] and abundant in cultured normal human keratinocytes and the HaCaT human epidermal cell line [11,52,53]. Thus, future studies are required to clarify the expression levels of *HAS1-3* mRNA in the normal human epidermis.

In summary, we provided evidence that the action of HAS3 in the production of epidermal HA may play a role in regulating epidermal morphogenesis by controlling keratinocyte proliferation and differentiation. These findings shed more light on our understanding of the functions of epidermal HA and its potential to regulate keratinocyte behavior.

## 4. Materials and Methods

### 4.1. Chemicals

The chemicals, 4-methylumbelliferone (4MU) and *Streptomyces* hyaluronidase (St-HAase) were obtained from Sigma-Aldrich (St. Louis, MO, USA).

### 4.2. Keratinocyte Cultures

Normal human skin keratinocytes from different donors (Kurabo, Osaka, Japan) were cultured in an MCDB153 medium (Wako Pure Chemical Industries, Osaka, Japan) containing 0.1 mM Ca^2+^ supplemented with 14.1 mg/L *o*-phosphorylethanolamine, 6.1 mg/L 2-aminoethanol, 5 mg/L insulin, 180 μg/L hydrocortisone, 100 ng/L epidermal growth factor, and 0.4% (*v*/*v*) bovine pituitary extract (Kurabo, Osaka, Japan) at 37 °C in a humidified atmosphere containing 5% CO_2_.

### 4.3. Reconstruction of Full-Thickness Human Skin Equivalents

Dermal equivalents were prepared using type I collagen solution (KOKEN, Tokyo, Japan) containing normal human dermal fibroblasts (Kurabo, Osaka, Japan). After four days of culture, keratinocytes were seeded onto the surface of the contracted collagen gel and cultured under submerged conditions for two days. When keratinocytes reached confluence, reconstructed skin equivalents were grown at the air–liquid interface and cultured for 9 days in the absence or presence of 0.1, or 1 unit/mL St-HAase or 0.5 mM 4MU. The epidermis was detached from the dermis using fine forceps.

### 4.4. RNA Interference

Knockdown experiments were conducted using Silencer^®^ Select siRNAs synthesized for *HAS3* (IDs: s6460 and s194496) and control non-silencing siRNA (Thermo Fisher, Waltham, MA, USA). These siRNAs were transfected into keratinocytes using Lipofectamine RNAiMAX (Thermo Fisher, Waltham, MA, USA), and the control and *HAS3* knocked-down cells were used for the reconstruction of epidermal equivalents.

### 4.5. Reconstruction of Human Epidermal Equivalents

Reconstructed human epidermal equivalents were prepared using keratinocytes transfected with *HAS3* siRNA or a control non-silencing siRNA. The cells were seeded on polycarbonate culture inserts (area of 0.6 cm^2^ with pores of 0.4 μm diameter; Millipore, Milford, KS, USA). After 24 h of incubation, the cells were exposed to the air–liquid interface and maintained in CELLnTEC Prime 3D Barrier Culture Medium (CELLnTEC, Bern, Switzerland) for 3–6 days.

### 4.6. Measurement of HA Content

Epidermal samples were obtained by punch biopsy from skin or epidermal equivalents and incubated with proteinase K (0.2 mg/mL) (Thermo Fisher, Waltham, MA, USA) in 0.1 M Tris-HCl (pH 7.4), 5 mM ethylenediaminetetraacetic acid, 0.4% (*w*/*v*) sodium dodecyl sulfate, and 0.2 M NaCl at 55 °C for 2 h. After heating at 95 °C, an equal volume of isoamyl alcohol/phenol/chloroform/(1: 25: 24, *v*/*v*/*v* ) was mixed and centrifuged to obtain the aqueous phase. The amount of HA was determined using the QnE HA ELISA Assay Kit (Biotech Trading Partners, Encinitas, CA, USA), according to the manufacturer’s protocol.

### 4.7. Quantitative Real-Time PCR

Total RNA was extracted from the skin or epidermal equivalents using an RNeasy Mini Kit (Qiagen, Hilden, Germany). cDNA was generated from the total RNA using a High-Capacity cDNA Reverse Transcription Kit (Applied Biosystems, Foster City, CA, USA). Target mRNA expression was quantitatively assessed using cDNA templates and a TaqMan real-time PCR assay (Applied Biosystems, Foster City, CA, USA) according to the manufacturer’s protocol. The relative quantification values of *HA synthase 1-3* (*HAS1-3*), *CD44*, *proliferating cell nuclear antigen* (*PCNA*), *involucrin* (*INV*), and *loricrin* (*LOR*) were normalized to endogenous *ribosomal protein lateral stalk subunit P0* (*RPLP0*). The data were analyzed using the ∆∆ quantitative C_T_ method [54].

### 4.8. Histological and Immunohistochemical Analysis

The skin or epidermal equivalents were fixed, paraffin-embedded, and sectioned. For morphological studies, the tissue sections were stained with H&E (Merck, Darmstadt, Germany). For immunofluorescence studies, sections were treated with mouse monoclonal anti-CD44 antibody (R&D Systems, Minneapolis, MN, USA), rabbit monoclonal anti-Ki67 antibody (Abcam, Cambridge, UK), mouse monoclonal anti-human filaggrin antibody (Biomedical Technologies, Tewksbury, MA, USA), mouse monoclonal anti-human involucrin antibody (Sigma-Aldrich, St. Louis, MO, USA), rabbit polyclonal anti-human loricrin antibody (Covance, Madison, WI, USA), or biotinylated HABP (HOKUDO, Hokkaido, Japan). The primary antibodies were detected with the following specific secondary antibodies: Cy3 or DyLight488-conjugated goat anti-rabbit IgG antibody for Ki67 (Jackson ImmunoResearch, West Grove, PA, USA), Cy3-conjugated goat anti-mouse IgG antibody for involucrin, filaggrin, and CD44 (Jackson ImmunoResearch, West Grove, PA, USA), DyLight488-conjugated goat anti-rabbit IgG antibody for loricrin (Jackson ImmunoResearch, West Grove, PA, USA), and streptavidin-fluorescein conjugates for biotinylated-HABP (Molecular Probes, Eugene, OR, USA). Immunofluorescence images were obtained using a fluorescent confocal microscope LSM710 (Carl Zeiss Microscopy, Jena, Germany). The epidermal thickness of H&E images was measured in 30 random positions per skin sample at ×200 magnification using the imaging software ZEN 3.1 (blue edition) (Carl Zeiss Microscopy, Jena, Germany).

### 4.9. Statistical Analysis

Statistical significance was assessed by Student’s *t*-test and Dunnett’s test using Microsoft Excel (Office 365) (Redmond, WA, USA) or IBM SPSS Statistics (version 25.0; IBM, Armonk, NY, USA). Statistical significance was set at *p* < 0.05.

## Data Availability

Not applicable.

## References

[1] Laurent T.C., Fraser J.R. (1992). Hyaluronan. FASEB J..

[2] Papakonstantinou E., Roth M., Karakiulakis G. (2012). Hyaluronic Acid: A Key Molecule in Skin Aging. Derm.-Endocrinol..

[3] Wang C., Tammi M., Tammi R. (1992). Distribution of Hyaluronan and Its CD44 Receptor in the Epithelia of Human Skin Appendages. Histochem. Cell Biol..

[4] Itano N., Kimata K. (1996). Expression Cloning and Molecular Characterization of HAS Protein, a Eukaryotic Hyaluronan Synthase. J. Biol. Chem..

[5] Shyjan A.M., Heldin P., Butcher E.C., Yoshino T., Briskin M.J. (1996). Functional Cloning of the cDNA for a Human Hyaluronan Synthase. J. Biol. Chem..

[6] Watanabe K., Yamaguchi Y. (1996). Molecular Identification of a Putative Human Hyaluronan Synthase. J. Biol. Chem..

[7] Weigel P.H., Hascall V.C., Tammi M. (1997). Hyaluronan Synthases. J. Biol. Chem..

[8] Rilla K., Siiskonen H., Spicer A.P., Hyttinen J.M.T., Tammi M.I., Tammi R.H. (2005). Plasma Membrane Residence of Hyaluronan Synthase Is Coupled to Its Enzymatic Activity. J. Biol. Chem..

[9] Sayo T., Sugiyama Y., Takahashi Y., Ozawa N., Sakai S., Inoue S., Ishikawa O., Tamura M. (2002). Hyaluronan Synthase 3 Regulates Hyaluronan Synthesis in Cultured Human Keratinocytes. J. Investig. Dermatol..

[10] Sayo T., Sakai S., Inoue S. (2004). Synergistic Effect of N-Acetylglucosamine and Retinoids on Hyaluronan Production in Human Keratinocytes. Ski. Pharmacol. Physiol..

[11] Kakizaki I., Itano N., Kimata K., Hanada K., Kon A., Yamaguchi M., Takahashi T., Takagaki K. (2008). Up-Regulation of Hyalu-ronan Synthase Genes in Cultured Human Epidermal Keratinocytes by UVB Irradiation. Arch. Biochem. Biophys..

[12] Sayo T., Sugiyama Y., Inoue S. (2013). Lutein, a. Nonprovitamin A, Activates the Retinoic Acid Receptor to Induce HAS3-Dependent Hyaluronan Synthesis in Keratinocytes. Biosci. Biotechnol. Biochem..

[13] Malaisse J., Bourguignon V., De Vuyst E., de Rouvroit C.L., Nikkels A., Flamion B., Poumay Y. (2014). Hyaluronan Metabolism in Human Keratinocytes and Atopic Dermatitis Skin Is Driven by a Balance of Hyaluronan Synthases 1 and 3. J. Investig. Dermatol..

[14] Matsui T., Amagai M. (2015). Dissecting the Formation, Structure and Barrier Function of the Stratum Corneum. Int. Immunol..

[15] Pasonen-Seppänen S., Karvinen S., Törrönen K., Hyttinen J.M., Jokela T., Lammi M., Tammi M.I., Tammi R. (2003). EGF Upregulates, whereas TGF-β Downregulates, the Hyaluronan Synthases Has2 and Has3 in Organotypic Keratinocyte Cultures: Correlations with Epidermal Proliferation and Differentiation. J. Investig. Dermatol..

[16] Karvinen S., Pasonen-Seppänen S., Hyttinen J.M., Pienimäki J.-P., Törrönen K., Jokela T.A., Tammi M.I., Tammi R. (2003). Keratinocyte Growth Factor Stimulates Migration and Hyaluronan Synthesis in the Epidermis by Activation of Keratinocyte Hyaluronan Synthases 2 and 3. J. Biol. Chem..

[17] Tammi R., Ripellino J.A., Margolis R.U., Maibach H.I., Tammi M. (1989). Hyaluronate Accumulation in Human Epidermis Treated with Retinoic Acid in Skin Organ Culture. J. Investig. Dermatol..

[18] Pasonen-Seppänen S.M., Maytin E.V., Törrönen K.J., Hyttinen J.M.T., Hascall V.C., MacCallum D.K., Kultti A.H., Jokela T.A., Tammi M.I., Tammi R.H. (2008). All-Trans Retinoic Acid-Induced Hyaluronan Production and Hyperplasia Are Partly Mediated by EGFR Signaling in Epidermal Keratinocytes. J. Investig. Dermatol..

[19] Rilla K., Pasonen-Seppänen S., Rieppo J., Tammi M., Tammi R. (2004). The Hyaluronan Synthesis Inhibitor 4-Methylumbelliferone Prevents Keratinocyte Activation and Epidermal Hyperproliferation Induced by Epidermal Growth Factor. J. Investig. Dermatol..

[20] Bourguignon L.Y., Ramez M., Gilad E., Singleton P.A., Man M.Q., Crumrine D.A., Elias P.M., Feingold K.R. (2006). Hyaluronan-CD44 Interaction Stimulates Keratinocyte Differentiation, Lamellar Body Formation/Secretion, and Permeability Barrier Homeostasis. J. Investig. Dermatol..

[21] Akazawa Y., Yoshida H., Endo Y., Sugita J., Yakumaru M., Sayo T. (2021). 1-Ethyl-β-N-Acetylglucosaminide Increases Hyaluronan Production in Human Keratinocytes by Being Converted to N-Acetylglucosamine via β-N-Acetylglucosaminidase-Dependent Manner. Biosci. Biotechnol. Biochem..

[22] Endo Y., Yoshida H., Akazawa Y., Yamazaki K., Ota Y., Sayo T., Takahashi Y. (2022). Antiwrinkle Efficacy of 1-Ethyl-β-N-Acetylglucosaminide, an Inducer of Epidermal Hyaluronan Production. Skin Res. Technol..

[23] Endo Y., Yoshida H., Ota Y., Akazawa Y., Sayo T., Hanai U., Imagawa K., Sasaki M., Takahashi Y. (2020). Accelerated Human Epidermal Turnover Driven by Increased Hyaluronan Production. J. Dermatol. Sci..

[24] Malaisse J., Pendaries V., Hontoir F., De Glas V., Van Vlaender D., Simon M., de Rouvroit C.L., Poumay Y., Flamion B. (2016). Hyaluronan Does Not Regulate Human Epidermal Keratinocyte Proliferation and Differentiation. J. Biol. Chem..

[25] Bourguignon L.Y. (2014). Matrix Hyaluronan-Activated CD44 Signaling Promotes Keratinocyte Activities and Improves Abnormal Epidermal Functions. Am. J. Pathol..

[26] Tammi R.H., Passi A.G., Rilla K., Karousou E., Vigetti D., Makkonen K., Tammi M.I. (2011). Transcriptional and Post-Translational Regulation of Hyaluronan Synthesis. FEBS J..

[27] Wang T.-P., Pan Y.-R., Fu C.-Y., Chang H.-Y. (2010). Down-Regulation of UDP-Glucose Dehydrogenase Affects Glycosaminoglycans Synthesis and Motility in HCT-8 Colorectal Carcinoma Cells. Exp. Cell Res..

[28] Kakizaki I., Kojima K., Takagaki K., Endo M., Kannagi R., Ito M., Maruo Y., Sato H., Yasuda T., Mita S. (2004). A Novel Mechanism for the Inhibition of Hyaluronan Biosynthesis by 4-Methylumbelliferone. J. Biol. Chem..

[29] Vigetti D., Rizzi M., Viola M., Karousou E., Genasetti A., Clerici M., Bartolini B., Hascall V.C., De Luca G., Passi A. (2009). The Effects of 4-Methylumbelliferone on Hyaluronan Synthesis, MMP2 Activity, Proliferation, and Motility of Human Aortic Smooth Muscle Cells. Glycobiology.

[30] Kultti A., Pasonen-Seppänen S., Jauhiainen M., Rilla K.J., Kärnä R., Pyöriä E., Tammi R.H., Tammi M.I. (2009). 4-Methylumbelliferone Inhibits Hyaluronan Synthesis by Depletion of Cellular UDP-Glucuronic Acid and Downregulation of Hyaluronan Synthase 2 and 3. Exp. Cell Res..

[31] Tamura R., Yokoyama Y., Yoshida H., Imaizumi T., Mizunuma H. (2014). 4-Methylumbelliferone Inhibits Ovarian Cancer Growth by Suppressing Thymidine Phosphorylase Expression. J. Ovarian Res..

[32] Lokeshwar V.B., Lopez L.E., Munoz D., Chi A., Shirodkar S.P., Lokeshwar S.D., Escudero D.O., Dhir N., Altman N. (2010). An-titumor Activity of Hyaluronic Acid Synthesis Inhibitor 4-Methylumbelliferone in Prostate Cancer Cells. Cancer Res..

[33] Urakawa H., Nishida Y., Wasa J., Arai E., Zhuo L., Kimata K., Kozawa E., Futamura N., Ishiguro N. (2012). Inhibition of Hya-luronan Synthesis in Breast Cancer Cells by 4-Methylumbelliferone Suppresses Tumorigenicity in Vitro and Metastatic Lesions of Bone in Vivo. Int. J. Cancer.

[34] Piccioni F., Malvicini M., Garcia M.G., Rodriguez A., Atorrasagasti C., Kippes N., Buena I.T.P., Rizzo M.M., Bayo J., Aquino J.B. (2011). Antitumor Effects of Hyaluronic Acid Inhibitor 4-Methylumbelliferone in an Orthotopic Hepatocellular Carcinoma Model in Mice. Glycobiology.

[35] Vigetti D., Rizzi M., Moretto P., Deleonibus S., Dreyfuss J., Karousou E., Viola M., Clerici M., Hascall V.C., Ramoni M.F. (2011). Glycosaminoglycans and Glucose Prevent Apoptosis in 4-Methylumbelliferone-Treated Human Aortic Smooth Muscle Cells. J. Biol. Chem..

[36] Kage M., Tokudome Y., Matsunaga Y., Hariya T., Hashimoto F. (2014). Effect of Hyaluronan Tetrasaccharides on Epidermal Dif-ferentiation in Normal Human Epidermal Keratinocytes. Int. J. Cosmet. Sci..

[37] Kage M., Tokudome Y. (2015). Hyaluronan Tetrasaccharides Stimulate Ceramide Production through Upregulated mRNA Expression of Ceramide Synthesis-Associated Enzymes. Arch. Dermatol. Res..

[38] Hascall V.C., Wang A., Tammi M., Oikari S., Tammi R., Passi A., Vigetti D., Hanson R.W., Hart G.W. (2014). The Dynamic Me-tabolism of Hyaluronan Regulates the Cytosolic Concentration of UDP-GlcNAc. Matrix Biol..

[39] Lee D.H., Oh J.-H., Chung J.H. (2016). Glycosaminoglycan and Proteoglycan in Skin Aging. J. Dermatol. Sci..

[40] Zimmermann D.R., Dours-Zimmermann M.T., Schubert M., Bruckner-Tuderman L. (1994). Versican Is Expressed in the Proliferating Zone in the Epidermis and in Association with the Elastic Network of the Dermis. J. Cell Biol..

[41] Qiu H., Liu F., Tao T., Zhang D., Liu X., Zhu G., Xu Z., Ni R., Shen A. (2016). Modification of P27 with O-Linked N-Acetylglucosamine Regulates Cell Proliferation in Hepatocellular Carcinoma. Mol. Carcinog..

[42] Andrés-Bergós J., Tardio L., Larranaga-Vera A., Gómez R., Herrero-Beaumont G., Largo R. (2012). The Increase in O-Linked N-Acetylglucosamine Protein Modification Stimulates Chondrogenic Differentiation Both in Vitro and in Vivo. J. Biol. Chem..

[43] Ogawa M., Mizofuchi H., Kobayashi Y., Tsuzuki G., Yamamoto M., Wada S., Kamemura K. (2012). Terminal Differentiation Program of Skeletal Myogenesis Is Negatively Regulated by o-GlcNAc Glycosylation. Biochim. Biophys. Acta.

[44] Ishihara K., Takahashi I., Tsuchiya Y., Hasegawa M., Kamemura K. (2010). Characteristic Increase in Nucleocytoplasmic Protein Glycosylation by o-GlcNAc in 3T3-L1 Adipocyte Differentiation. Biochem. Biophys. Res. Commun..

[45] Delehedde M., Lyon M., Sergeant N., Rahmoune H., Fernig D.G. (2001). Proteoglycans: Pericellular and Cell Surface Multireceptors that Integrate External Stimuli in the Mammary Gland. J. Mammary Gland Biol. Neoplasia.

[46] Perrot G., Colin-Pierre C., Ramont L., Proult I., Garbar C., Bardey V., Jeanmaire C., Mine S., Danoux L., Berthélémy N. (2019). Decreased Expression of GPC1 in Human Skin Keratinocytes and Epidermis during Ageing. Exp. Gerontol..

[47] Ojeh N., Hiilesvuo K., Wärri A., Salmivirta M., Henttinen T., Määttä A. (2008). Ectopic Expression of Syndecan-1 in Basal Epidermis Affects Keratinocyte Proliferation and Wound Re-Epithelialization. J. Investig. Dermatol..

[48] Bai K.-J., Spicer A.P., Mascarenhas M.M., Yu L., Ochoa C., Garg H.G., Quinn D.A. (2005). The Role of Hyaluronan Synthase 3 in Ventilator-induced Lung Injury. Am. J. Respir. Crit. Care Med..

[49] Kobayashi N., Miyoshi S., Mikami T., Koyama H., Kitazawa M., Takeoka M., Sano K., Amano J., Isogai Z., Niida S. (2010). Hyaluronan Deficiency in Tumor Stroma Impairs Macrophage Trafficking and Tumor Neovascularization. Cancer Res..

[50] Mack J.A., Feldman R.J., Itano N., Kimata K., Lauer M., Hascall V.C., Maytin E.V. (2012). Enhanced Inflammation and Accelerated Wound Closure Following Tetraphorbol Ester Application or Full-Thickness Wounding in Mice Lacking Hyaluronan Synthases Has1 and Has. J. Investig. Dermatol..

[51] Gariboldi S., Palazzo M., Zanobbio L., Selleri S., Sommariva M., Sfondrini L., Cavicchini S., Balsari A., Rumio C. (2008). Low Molecular Weight Hyaluronic Acid Increases the Self-Defense of Skin Epithelium by Induction of β-Defensin 2 via TLR2 and TLR. J. Immunol..

[52] Averbeck M., Gebhardt C.A., Voigt S., Beilharz S., Anderegg U., Termeer C.C., Sleeman J., Simon J.C. (2007). Differential Regulation of Hyaluronan Metabolism in the Epidermal and Dermal Compartments of Human Skin by UVB Irradiation. J. Investig. Dermatol..

[53] Rauhala L., Jokela T., Kärnä R., Bart G., Takabe P., Oikari S., Tammi M.I., Pasonen-Seppänen S., Tammi R.H. (2018). Extracellular ATP Activates Hyaluronan Synthase 2 (HAS2) in Epidermal Keratinocytes via P2Y2, Ca2+ Signaling, and MAPK Pathways. Biochem. J..

[54] Schmittgen T.D., Livak K.J. (2008). Analyzing Real-Time PCR Data by the Comparative C(T) Method. Nat. Protoc..

